# Moderation of Physical Health on Emotional Health Among Grandparents Raising Grandchildren

**DOI:** 10.1093/geroni/igaa057.1483

**Published:** 2020-12-16

**Authors:** Yuqin Jiao, Nathaniel Riggs, Loriena Yancura, Aimee Fox, Christine Fruhauf

**Affiliations:** 1 Colorado State University, Fort Collins, Colorado, United States; 2 University of Hawai’i, Honolulu, Hawaii, United States

## Abstract

An estimated 69.5 million older adults in the United States report being grandparents. It is also evident that the number of grandparents raising grandchildren among them is increasing. Although caring for their grandchildren often provides grandparents a sense of purpose and increased life satisfaction, unexpected responsibilities of parenting worsen the already challenging experience of aging (e.g., potential for poor physical and mental health). Thus, it is essential to study the well-being of grandparents raising grandchildren. Links between depressive symptoms and emotional health have been widely studied in previous research. However, limited research has focused on physical health as a potential moderator, despite indirect evidence shown that more depressive symptoms may be observed among those with worse physical health conditions. To address this, we used Optum® SF-36v2® Health Survey to collect physical- and mental-health data and Center for Epidemiological Studies Depression Scale (CES-D-10) to collect information on depressive symptoms in 137 grandparents raising grandchildren (age 40-83) before, immediately after, and six months after a six-week intervention focused on self-care practices. The presence of more depressive symptoms indicated worse emotional health. Physical health moderated these associations at all three time points, such that depressive symptoms were less strongly related to emotional health if grandparents self-reported better physical-health scores. These findings have important implications for future intervention studies. The importance of good physical health practices for grandparents raising grandchildren is evident, including increased physical engagement and better pain management.

